# Public acceptance of policy measures to prevent and combat infectious diseases in pets: a systematic literature review

**DOI:** 10.3389/fvets.2025.1603592

**Published:** 2026-01-12

**Authors:** Michelle Kim Emily Wyler

**Affiliations:** KPM Center for Public Management, University of Bern, Bern, Switzerland

**Keywords:** acceptance, companion animals, policy measures, public health, zoonoses

## Abstract

Zoonotic diseases represent a critical and growing threat to global public health. While farm and wild animals are already heavily integrated into policy measures of disease prevention, companion animals do often not get included in the fight against zoonotic diseases, despite their proximity to humans. Nevertheless, policy measures focusing on household pets are a crucial element in the prevention and combat of infectious diseases. This systematic literature review, following the Preferred Reporting Items for Systematic Reviews and Meta-Analyses (PRISMA) guidelines, summarizes the research on pet-targeted disease prevention measures, focusing on factors influencing their acceptability. Factors influencing acceptance were categorized in an expanded Nodality-Authority-Treasure-Organization (NATO) scheme adapted from Hood. A total of 95 articles were included in the study and provide insights across thirty-two countries. Thereby, personal owners' characteristics emerge as the most frequently studied factor influencing acceptance. The review found information provision by veterinarians, affordability, organizational accessibility, and pet controllability to be the most influential factors affecting public acceptance. This review highlights that the interplay of policy decisions with factors of context must be considered in order to accelerate acceptance, and that successful implementation depends on integrating veterinary, social, and behavioral perspectives.

## Introduction

The World Health Organization (WHO) estimates that 60% of human pathogens originate from animals. Furthermore, 75% of pathogens causing emerging and reemerging animal diseases possess the potential to cross the animal-human barrier (1). Therefore, the rise of zoonotic diseases presents a complex global challenge that requires a collaborative and interdisciplinary approach, also including the scientific community.

One Health involves balancing and optimizing the wellbeing of humans, animals, and the wider environment. The concept acknowledges that the health of humans, wild and domestic animals, and the environment are connected and reliant (2). Zoonotic disease prevention is an important intersection where a One Health approach is crucial. As a result of zoonotic outbreaks, such as mad cow disease (bovine spongiform encephalopathy), avian influenza, and swine fever (swine acute diarrhea syndrome), the need for research on effective measures to combat diseases in animals has been thematized worldwide.

However, while zoonotic diseases in connection with livestock have already drawn considerable attention in policymaking (3, 4), a gap can be identified in the area of household pets, i.e., animals which are living with humans in households as companions, for example, cats (*Felis catus*) and dogs (*Canis familiaris*), despite the epidemiological roles of pets: Pets can serve as early warnings for human disease outbreaks (e.g., West Nile virus in birds, Lyme disease in dogs); facilitate spillovers from wildlife to humans due to their intermediate position (e.g., ticks on pets bringing Lyme disease into houses); or maintain and amplify zoonotic cycles (e.g., rabies in dogs, Bartonella in cats). The COVID-19 pandemic, in which cats and, to a smaller extent, dogs have also been carriers of the disease (5, 6), has emphasized the significance of research and policy actions to address the risks of zoonotic diseases in connection with pets, as rabies had before. As further disease outbreaks originating from zoonoses can be expected in the future, it is essential to appropriately address these public health threats, including measures targeting livestock, wild, and companion animals.

This raises the question of whether policy measures for companion animals must differ from those for livestock and wild animals. Pets have a unique status in modern society. For many people, a pet is a member of the family to which they build a deep emotional attachment; pets have a higher emotional value in society and social function than other animals (7, 8). This forms a requirement for special sensitivity when developing socially acceptable measures for pets in the context of epidemics.

Public policy literature states that the acceptance of policy measures is crucial to ensure their successful implementation (9, 10). Consequently, animal disease prevention and control measures that are implemented without public support are less likely to be effective. Since companion animals occupy a special position in modern societies, it can be supposed that disease prevention measures addressing pets also have to overcome different barriers in order to be socially acceptable.

Global warming suggests an increased incidence of infectious diseases in the future (11). Therefore, a closer consideration of pets in disease prevention and control policies is necessary in order to safeguard global health and prepare for future outbreaks of infectious diseases. Consequently, it is essential to attain insights into which policy measures are perceived by stakeholders, i.e., pet owners, animal rights activists, bureaucrats and veterinarians, the private sector, and the general public, as acceptable, in order to design suitable policy measures and thus create new perspectives for pet owners as well as their animals to be prepared for and adequately treated during future pandemics. In order to comprehend public acceptance of individual measures, it is essential to understand the drivers and barriers of acceptance. Despite the described relevance of the topic, no systematic literature review exists in the area of infectious disease measures targeting household pets or factors influencing their acceptance from the society. In order to close this gap, the present study provides an overview of existing research on zoonotic pet disease control measures, including categorization of regions, diseases, stakeholders, and animals researched, before focusing on the factors influencing their acceptance. In this regard, the study seeks to respond to the question: *What factors influence the acceptance of and resistance toward disease prevention and control measures for household pets*?

This study provides new perspectives for researchers, policymakers, and practitioners on developing strategies to implement and promote infectious disease measures that are socially accepted. Furthermore, this study helps to satisfy the needs of pet holders to be addressed and considered in a satisfactory manner in disease prevention measures targeted at their pets.

## Methods

A comprehensive literature search, in the form of a systematic review of existing research on public acceptance of policy measures targeting infectious diseases in household pets, was performed. Public policy measures for the prevention and combat of zoonoses in companion animals were identified, and their acceptance levels among stakeholders and factors influencing acceptance and resistance investigated. This study was conducted by following the reporting checklist of the Preferred Reporting Items for Systematic Reviews and Meta-Analyses (PRISMA) (12). Ethical approval was not required for this review, as the data are publicly available.

### Data sources and search strategy

The search was conducted on three databases in October 2023: Scopus, Web of Science, and Google Scholar. The choice of the mentioned databases lies in their data size and scientific diversity. No limitations were placed on the publication date. The languages of the publications were limited to English, French, German, and Spanish. The search strategy for the three databases was as follows:

Scopus
(TITLE-ABS-KEY (pet OR pets OR companion AND animal^*^) AND TITLE-ABS-KEY (disease^*^ OR zoono^*^ OR epidemic^*^ OR virus) AND (infect^*^ OR spread^*^) AND TITLE-ABS-KEY (method^*^ OR policy OR policies OR strateg^*^ OR combat^*^ OR success OR strateg^*^ OR procedure^*^ OR measure^*^) AND NOT (polyethylene AND terephthalate) AND NOT (positron AND emission AND tomography) AND NOT (primary AND economic AND terms) AND NOT (practice AND question AND evidence AND translation))
Web of Science
TS=(pet OR pets OR companion animal) AND TS=(diseas^*^ OR zoono^*^ OR epidem^*^ OR virus) AND TS=(infect^*^ OR spread^*^) AND TS=(method^*^ OR policy OR policies OR strateg^*^ OR combat^*^ OR success OR procedure^*^ OR measure^*^) NOT TS=(polyethylene AND terephthalate) NOT TS=(positron AND emission AND tomography) NOT TS=(primary AND economic AND terms) NOT TS=(practice AND question AND evidence AND translation) AND LA=(English OR German OR French OR Spanish)
Google Scholar
pet|“companion animal” disease|zoonosis|zoonotic infection|spread method|policy|strategy|combat|success|measure|procedure -“polyethene terephthalate” and -“positron emission tomography” -“primary economic terms” -“practice question evidence translation”

The terms “polyethene”, “terephthalate”, “positron”, “emission”, and “tomography” were excluded in order to remove unwanted articles referring to studies on acronyms of PET with no relation to pets as companion animals.

### Study selection and data extraction

The search of three databases resulted in 6,425 initial hits. The acquired publications were merged into a Microsoft Excel file for the selection process. In a first step, titles and abstracts were assessed to evaluate eligibility, and non-conforming publications were removed. Duplicates originating from the searches of different databases were manually removed. To prevent omissions, from the 49 remaining articles, all references were searched, and the respective literature was equally reviewed for eligibility. In the following, the final eligibility was determined by full-text reviews. With the help of a second researcher, uncertainties regarding the inclusion of articles were discussed and resolved.

Inclusion criteria were that the publications focused on one or more stakeholders [owners, general public, veterinarians, public health workers, community leaders, private sector businesses, state agencies, activists, international organizations, children, scientists] and on one or more disease prevention or combat measures. Pets in general, or one or several animals of the sort of household pets [dogs (*Canis familiaris*), cats (*Felis catus*), bunnies/rabbits (*Oryctolagus cuniculus domesticus*), equines, birds (ornamental birds, pet ducks, pet hens & pet roosters (*Gallus domesticus*)), herpetofauna, geckos, reptiles, amphibians, alligators (*Alligator mississippiensis*), tortoises, axolotl (*Ambystoma mexicanum*), hamsters, ferrets, guinea pigs (*Cavia porcellus*), pet raccoons, pet hedgehogs, mice, gerbils, rats, ornamental fish, pet pigs, hobby sheep (*Ovis aries*), goats (*Capra hircus*)], were to be mentioned as subject to a disease prevention or combat measure. Quantitative, qualitative, and conceptual studies were included. Publications with a different focus but relevant data to the research question were included.

Editorials, opinion pieces, news articles, governmental publications, guidelines, and non-peer-reviewed articles were excluded.

### Data analysis, synthesis, and presentation

All included studies were carefully reviewed to extract and code the data.

A synthesis of all reviewed articles was performed, and a structured framework with the following subgroups was designed:

(i) Countries and areas covered
(ii) Companion animals
(iii) Diseases
(iv) Stakeholders
(v) Disease prevention and combat measures
(vi) Factors influencing acceptance of policy measures

Supplementary Table 1 shows the included studies and their characteristics.

Data of subgroups i, ii, and iii were compared and analyzed to extract general tendencies in studies conducted. Subgroup v was further categorized, and the measures were grouped into lists of sub-measures. Subgroup vi was transformed into a table of positive, negative, and neutral factors and grouped into a NATO scheme adapted from Hood (13). Subgroups iv and v are integrated into the table.

The NATO anagram refers to typologies of policy instruments and details the following: *Nodality* describes policy instruments with the core idea of information collection and release, with advice-giving and advertising as the main functions. The category of *authority* centers around regulations, laws, standard settings, committees, and consultations, for example, in the form of laws and decrees. *Treasure* is the third category and describes instruments relying on governmental resources and funds, regulating financial support and sanctions. Finally, *organization-*based policy instruments tackle the direct provision of goods and services and create markets for the said purpose (13, 14).

Besides the described categories, three additional subgroups have been added to the NATO scheme in order to better integrate external factors influencing policy measures. The *owner characteristics* describe aspects of the acceptance subject influencing policy acceptance. *Pet characteristics* center around attributes of pets targeted by measures. The last category, *context*, contains components relating to the background of policy measures, such as the severity of the disease, duration of treatment, or additional effects of medicines. The NATO scheme was developed by Hood in 1983 and is used to analyse policy choices and develop recommendations.

### Quality assessment

To assess the methodological quality of the included studies, the Joanna Briggs Institute (JBI) critical appraisal checklist (15), which provides design-specific tools for evaluating the risk of bias, was used. Given the diversity of the study types included in this review (quantitative, qualitative, and conceptual studies), the JBI checklists for cross-sectional, qualitative, and mixed-methods research were used. One reviewer assessed the studies to ensure consistency, and any uncertainties were discussed with a second researcher until a consensus was reached. The application of the JBI checklists indicated variations in methodological quality across the 95 included studies. These observations were considered when interpreting the findings.

## Results

### Literature search

**Figure 1:**
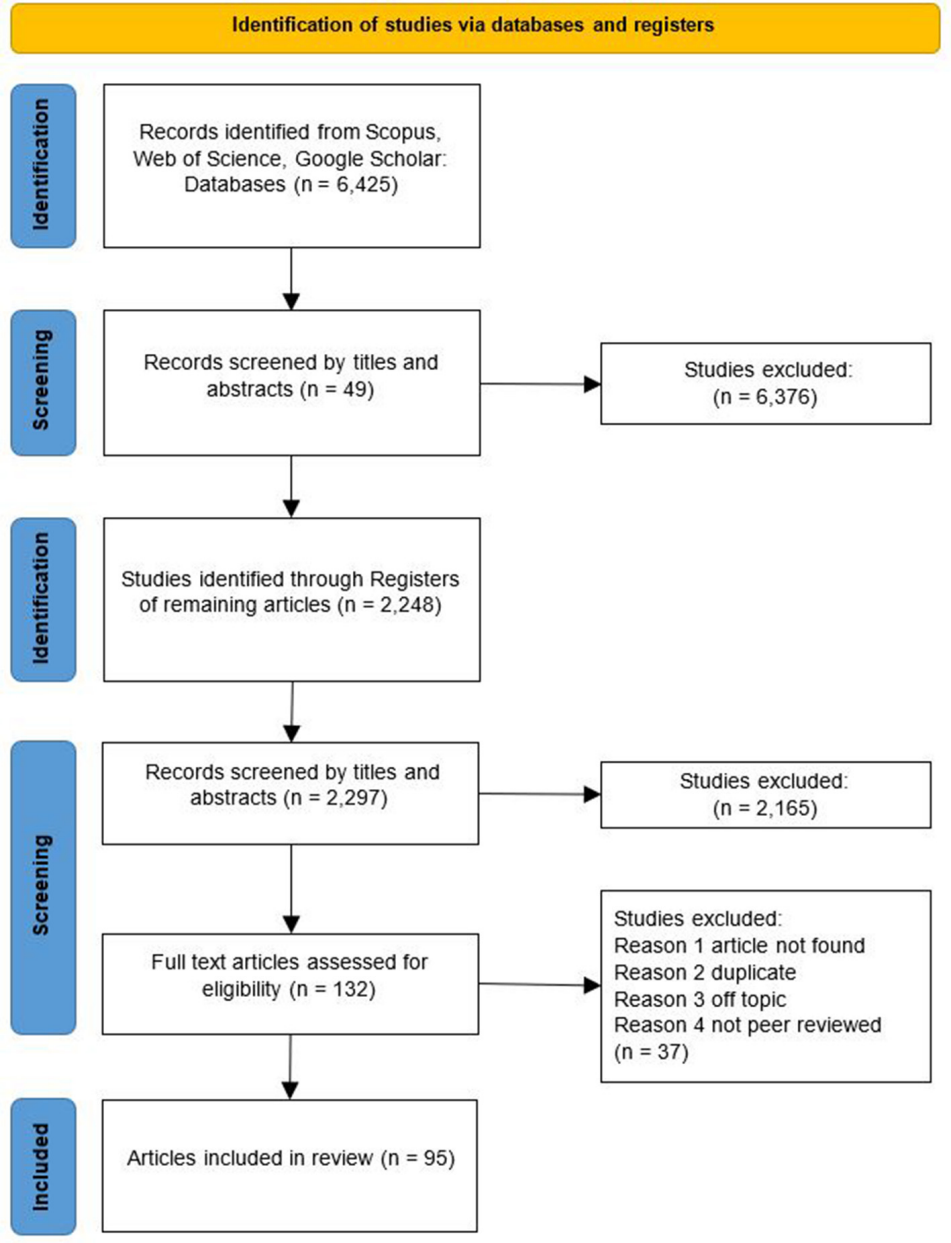
PRISMA flow diagram of literature research.

Figure 1 shows the processes used to filter the literature using the PRISMA checklist (12). The search yielded 95 articles. These articles were conceptual as well as empirical studies, and the empirical studies were conducted both qualitatively and quantitatively. The timeframe of the included articles ranged from 1950 to 2023 and is shown in Supplementary Table 2.

### Characteristics of the included studies: regions, animals, and diseases

The 95 analyzed articles deal with a total of 32 different countries. In Table 1, the covered countries are listed with referenced articles.

**Table 1:** Country distribution of included studies.

**Context**	**No. of countries in context covered by articles**	**Article references**
African countries	5	(57–69)
Asian countries	13	(16, 70–85, 101–104)
Western countries	11	(17–47, 86–94, 98–100, 105, 106, 109–111)
South America / Caribbean countries	4	(95–97, 107, 108)
Total	33	

The country to which most studies refer is the United States of America (16–41), with 27 studies, and the second most targeted country is Australia (31, 42–47) with 7 articles. 8 articles were not country-specific (48–55).

In Table 2, articles covering countries sorted by their economic status are listed, whereby country classification follows the understanding of the World Trade Organization (56).

**Table 2:** Research countries' economic status of included studies.

**Context**	**No. of articles**	**Article references**
Developing countries	34	(53, 57, 59–69, 71, 73, 76–81, 84, 85, 95, 96, 101, 103, 104, 107, 108)
Industrialized countries	60	(16–47, 70, 71, 74, 86–94, 98–100, 102, 106, 109–111)
Total	94	

The majority of articles focus on dogs (16, 18, 19, 22–24, 28, 31, 32, 34, 35, 48–50, 53–55, 57–97) as the target animal of infectious disease measures, in a total of 61 articles. The second largest category of covered animals refers to cats (16, 22–24, 26, 27, 30, 34, 35, 43, 49–51, 53–55, 72, 74, 81, 86, 89–91, 93, 96, 98) with 29 articles, and the third largest category looks at equines with 10 articles (20, 44–47, 50, 54, 55, 99, 100). 16 articles did not specify any particular animal (25, 29, 38–42, 52, 101–108).

The analyzed studies look at a large number of different zoonoses. However, by far the most covered disease is rabies, researched in 39 articles (16, 22, 23, 49, 57–71, 73, 75–87, 89, 94–97, 103). A total of 38 articles are not disease-specific (18–20, 24–27, 29–31, 38–43, 51, 52, 54, 55, 74, 91, 93, 98–102, 104, 105, 107, 109, 110).

All 13 studies conducted in African countries focus on dogs and rabies (57–69). A total of 21 studies conducted in Asian countries research the disease rabies (16, 70, 71, 73, 75–85, 103) or CoV-19 (16, 71, 72, 104) or stay as not disease-specific (74, 101, 102, 104), while focusing on dogs, cats, or remaining non-specific about the researched companion animal. Meanwhile, studies conducted in Western countries are not only more numerous, with 48 studies being conducted (17–47, 86–94, 98–100, 105, 106, 109–111), but also include a wider field of research on diseases and companion animals.

A total of 11 different stakeholders were identified in the articles: Pet owners, veterinarians, public health workers, animal rights activists, children, community leaders, state agencies, international organizations, scientists, the private sector, and the general public. Table 3 shows the distribution of articles among the stakeholder groups.

**Table 3:** Stakeholder focus of included studies.

**Stakeholder**	**No. of articles**	**Article references**
Owners	61	(17, 19–22, 26–28, 30, 31, 33–36, 42–46, 53, 57, 59–69, 71, 73, 74, 76, 77, 79–84, 86, 88, 89, 91–93, 95–98, 101, 102, 107–111)
Veterinarians	26	(18, 21, 23, 29, 32, 34, 35, 38–42, 47, 50–53, 71, 86, 93, 94, 99, 100, 105–107)
Others	39	(16–18, 21, 23–25, 31, 36, 37, 48, 49, 54, 55, 58, 63, 64, 68–73, 75, 76, 78, 79, 81, 85–87, 90, 94, 96, 98, 103, 104, 106, 107)
Total	126	

There were no anomalies or irregular distributions by region, animal, or disease with regard to the stakeholders.

### Measures to combat infectious diseases in companion animals

A total of 288 measures to combat infectious diseases in companion animals were identified in the data, including forms of animal population control, biosecurity practices, communication outreach to the general population and education/information campaigns, disease vector control campaigns, the elaboration of guidelines, immunization, legislation, reporting, surveillance, and testing. The most cited measure in combating infectious diseases in household pets is vaccination, which consisted in a research focus in 51 articles (16, 17, 19, 20, 22–24, 26, 27, 30, 36, 37, 43, 46, 48–51, 57–70, 73, 74, 76, 77, 79, 80, 82–84, 86, 87, 89, 94–98, 103, 110) and covers a wide spectrum regarding both animal species and illnesses. However, a focus on rabies prevention for dogs can be observed in regard to this measure.

### Factors influencing acceptance

From 95 analyzed articles, 67 include factors influencing acceptance of infectious disease measures (16, 17, 19–22, 25–28, 30–32, 34–39, 43–47, 53, 57–73, 75, 77–79, 82–84, 86, 88–90, 92–98, 100, 102, 104, 105, 108–110). Extracted information on factors influencing the acceptance and resistance of measures to prevent and combat infectious diseases in pets is shown in Supplementary Table 1. An adapted version of the NATO scheme according to Hood (13) has been used to categorize the factors, with the addition of three categories: owner characteristics, pet characteristics, and context, in order to include aspects not fitting in nodality, authority, treasure, or organization.

### Nodality

*Information distribution* is the most mentioned influencing factor in the category of nodality. Hereby, *information distribution and explanation by veterinarians* (17, 19, 26, 27, 30, 34, 36, 44, 45, 73, 90, 92, 93), for example during the examination of a patient or by the display of flyers or posters in the veterinary cabinet, was found to be the most important factor positively influencing acceptance, and *information distribution through television, radio, and national newspapers* (72, 73, 78, 79, 90, 93, 94) was the second most cited factor positively influencing acceptance. Further tools for information distribution include *text message alerts, letters, telephone calls, megaphone/sound truck alerts*, and *town criers* (57, 73, 79, 94, 95, 97), all of which were pointed out as positively influencing the acceptance of vaccination. In the mentioned studies, the acceptance subjects received personalized messages, letters, or telephone calls, informing them about vaccine campaigns taking place in their close surroundings. Megaphones, sound trucks, and town criers, on the other hand, were impersonalised tools, circulating neighborhoods and informing about soon-to-take-place vaccination campaigns.

*Incomplete information/lack of information* and *misinformation* (31, 37, 39, 44, 59–62, 65, 73, 89, 93, 95, 96) (e.g., outdated information or conspiracy theories) are the most frequently listed negative influencing factors, affecting a total of thirteen different policy measures. A related factor, *miscommunication* (21, 44, 62, 95) (e.g., sending information messages with bad timing or threatening content), was shown to have a negative impact on the acceptance of vaccination and biosecurity practices. *Emotional media coverage*, such as footage of crying children mourning for their pets, was shown to be an acceptance-reducing factor on culling (86), as were *actors from the scientific community* or the *WHO communicating that they are not in favor of the measures* (16).

Nodality tools that were shown to have no influence on public acceptance of measures stood in relation to the measures culling and seizure of pets: One study conducted in an authoritarian regime concluded that *state officials calling out resistance to measures* and *state officials and scientists praising measures* (86) had no effect on the public acceptance of the aforementioned measures.

### Authority

Regarding tools of authority, *strong and early leadership* on a governmental as well as on a local level showed a strong influence on the acceptance of policy measures (37, 47, 57, 89). For example, the placement of liability on pet owners and the consequent enforcement of laws turned out to be a main factor in higher vaccination participation.

*Restrictive jurisdictional and regulatory frameworks*, on the other hand, were pointed out to have a negative impact on public acceptance of a total of 14 measures (37). The creation of laws such as *anti-neglect laws* and *laws which impose higher or minimum standards for the treatment of companion animals*, i.e., laws which support the singular animal's rights and grant more protection against abuse, were shown to have a negative impact on the acceptance of measures such as the seizure and the abandonment of pets (16, 86).

### Treasure

Concerning the category treasure, the acceptance of policy measures was shown to be positively related to the *affordability* of measures, such as a cheap price of a medicament or a vaccination, and to *incentives* given for participation, such as bracelets for the pet owner or collars for the pet, *financial support* for companies and *free participation* (46, 57, 60, 92, 104).

In a total of 17 articles, the *absence of money/financial support* is mentioned as a reduction in policy measure acceptance (19, 22, 30, 34, 37, 61, 62, 65, 67–70, 77, 86, 88, 89, 105). The acceptance of vaccination is mentioned to be negatively influenced by *costs/higher prices* in 13 different studies (19, 22, 30, 37, 61, 62, 65, 67–70, 77, 89).

### Organization

Organizationally, *long waiting times* and *unideal opening hours*, for example, during school hours or while farmers are away from town, showed a negative influence on acceptance of policy measures (26, 27, 34, 62, 64, 96). Furthermore, an *inadequate location* of the vaccination point, such as a far distance to the home of pet owners or security issues in the surroundings, showed a negative influence on the measure vaccination in the highest number of articles (26, 31, 34, 59, 62, 66, 69, 77, 95, 96).

On the other hand, *easy access policy measure* had a positive influence on the acceptance of measures (44, 57, 58, 68). Furthermore, *population inclusion*, such as volunteer programmes for the implementation of measures, or citizen consultation during development, showed a positive influence on the largest number of different measures (61, 82).

### Owner characteristics

The owner characteristics category, i.e., defining aspects of the human being in charge of the decision if a measure is to be accepted, comprises the most factors mentioning a positive influence on acceptance of disease prevention measures for pets. *General interest in the disease* or *experience with disease cases* (17, 47, 92, 93), as well as *frequent*^1^ *travelling with a companion animal* (19, 22, 27) are shown to positively influence the acceptance of measures. *Awareness/strong risk perception/fear of disease* is shown to positively influence the acceptance of 26 different policy measures (32, 34, 36, 38, 46, 47, 72, 98, 105, 109) such as culling, vaccination, and the implementation of biosecurity practices.

*Unawareness of disease*, on the contrary, is also frequently described as a strong indicator of lower acceptance of measures (28, 59, 62, 65, 73, 78, 89, 95, 96). However, *unawareness of the disease* showed a positive influence on the acceptance of traditional medicines and spiritual practices, such as cures with holy water (58). *Concerns about adverse reactions* to vaccination are shown to lower the acceptance of vaccination in 9 studies (19, 26, 27, 30, 58, 62, 77, 93, 95). Furthermore, the *existence or absence of an animal care culture* also shows influence on the acceptance of the population toward vaccination, displaying a higher acceptance of vaccination in communities with an animal care culture (79, 95).

The *perception* of veterinarians and related professions that *clients would gain a positive picture of them by seeing them applying the disease prevention measures* shows a positive influence on their acceptance toward said measures positively (25, 47). On the other hand, the *fear of a negative client reaction* (38, 47, 93) shows to lower veterinarians acceptance of policy measures such as preventive medicine and wearing personal protective equipment (PPE). Also, *fear of being perceived in a negative light by their colleagues*, i.e. *the vet culture* in which an “overly protective” or “frightful” approach is disregarded, is pointed out as lowering veterinarians' acceptance of measures such as PPE (38, 47, 105). Furthermore, *working in a private veterinary clinic* is shown as standing in a relationship to a lower acceptance of disease prevention measures (47, 100, 105).

Aspects such as *age* (46), *sex* (27, 44, 63, 96), and *religion* (59, 63, 66, 79) of owners and the community are researched in different articles with contradicting results.

### Pet characteristics

All relevant pet characteristics elaborated throughout the review are related to dogs, cats, and equines. The most mentioned positive influencing factor on acceptance in relation to concerned animals was the *purpose of the pet*, such as working dogs for hunting or guarding (19, 62, 79). In this regard, pets with utilitarian value have a higher chance of being vaccinated. *Purebred pets/pets from a breeder* also show to stand in relationship with a higher acceptance of measures such as vaccination (26, 27).

The most mentioned factor negatively influencing acceptance of, for example, vaccination, is shown to be *hard-to-handle pets*, such as aggressive dogs or stressed cats (30, 59–62, 65, 77). Furthermore, *female pets*, due to their lower value in certain cultures, are shown to be less frequently vaccinated than their male counterparts and more frequently abandoned (79, 83, 104).

### Context

In the last category on the wider context of disease prevention measures for pets, *safety* and *animal welfare ratings* of the individual measures and their *compliance with industry guidelines* and *animal regulations* are shown to increase acceptance of respective measures (22, 39, 105).

“Technical applications”, for example, mobile applications for diagnosing pet diseases or applications for surveillance systems, have shown a higher acceptability if they disposed of *user-friendliness* and *technical quality* (71, 101, 102). Issues with *data confidentiality* are shown to lower their acceptance (21).

The *severity of the disease, existence of human fatal cases* and an *early stage of the disease outbreak* also show to have a positive influence on an public acceptance of different measures, such as vaccination, biosecurity measures and culling (26, 46, 47, 86).

*Areas of recent dog culling* are shown to stand in a negative relation to public acceptance of the measure vaccination (83, 84).

## Discussion

### Summary of principal findings

A total of 95 articles were analyzed to identify the factors shaping public acceptance of policy measures aimed at preventing and controlling infectious diseases in companion animals. The review includes published peer-reviewed articles and excludes government reports and other unpublished sources. This review demonstrates that acceptance is influenced by an interplay of informational, financial, organizational, and socio-cultural factors, with veterinarians emerging as trusted actors in communicating and legitimizing disease prevention measures. The most researched animals in analyzed articles were dogs, the most researched disease was rabies, and the most researched stakeholder group was pet owners. A far greater variety of researched diseases can be observed in the context of industrialized countries compared to developing countries. This appears paradoxical, since not only the disease burden but also the diversity of infectious diseases is highest in sub-Saharan Africa (112). While vaccination showed to be the most researched measure, a number of measures (e.g. leash laws, pet curfews, containment) are aimed at reducing contact between pets and wildlife (breaking zoonotic cycles) and can thus be considered One Health approaches. Information distribution, not only by veterinarians but also through the media, has been shown in a diverse range of studies as a powerful tool to increase acceptance of disease control measures in pets. Pet owners react with higher levels of acceptance to a policy measure targeting their pets if the measure is explained and backed by the support of veterinarians. This stands in line with the high level of trust pet owners generally hold in veterinarian advice and care (113). Equally, direct modes of transmitting information, such as text messages, letters, telephone calls, megaphone and sound truck alerts, and engaged town criers, show a positive influence on public acceptance. Conversely, the absence of information distribution, confusing information, or misinformation show a clear connection to lower public acceptance of a range of policy measures. These conditions can be provoked, for example, by a very rural environment, long distances between public and health institutions, or a strong presence of religious or tribal beliefs regarding diseases or animals. Regarding authority, strong and early leadership shows to positively influence the acceptance of policy measures. Such leadership could take the form of a government institution reaching out to the population through a press conference. It could also appear in the role of a local village chief leading the population through a vaccination campaign. The creation of laws and standards for the ethical treatment of animals is demonstrated to lower the public acceptance of measures that resulted in the death of pets. However, the variability in approaches to policy measures that different countries apply, as well as the political, social, and economic specificities of regions, must be considered when interpreting those results.

Fiscal aspects are generally confirming lower prices, free access, samples, and incentives to support the acceptance of measures, while higher prices and poverty tend to be lowering factors.

In organizational terms, a bad location of central facilities, long waiting times, and unideal opening hours are shown to lower acceptance of policy measures. Easily accessible policy measures with low time commitment and bureaucratic effort show a positive influence on acceptance of the measure. Population inclusion and citizenship involvement in policy measures and their execution are exhibited to have a positive influence on the largest number of measures.

Personal characteristics leading to (un-)acceptance of measures are discussed extensively in a large number of studies. The results show that general interest in the disease, experience with concrete disease cases, and the related characteristic of a stronger risk perception and awareness have a positive influence on acceptance of disease prevention measures, while unawareness lowers it. Worth mentioning in relation to the measure vaccination is the positive influence on acceptance frequent travel habitudes show, while concerns about adverse reactions are pointed out to lower acceptance.

A special focus can be placed on the attributes of veterinarians, regarding their acceptance of disease prevention measures, whereby the perceptions of their clients and colleagues seem to have a strong influence on veterinarians. Furthermore, veterinarians working in private clinics tend to have a lower acceptance of disease prevention measures than those working in public and shared clinics and teaching hospitals.

Regarding pet characteristics, animals serving a concrete purpose and animals obtained from a breeder are characteristics positively related to higher acceptance of measures such as vaccination. In other words, the perceived value of the pet stands in direct relation to the acceptance of its vaccination.

Hard to handle pets which cause problems on the way to or during the measure; for example, through fights with other pets, biting, or being uncatchable, as well as female animals are less likely to be vaccinated, leashed, or tested, and more frequently abandoned. The findings of the personal and animal characteristics sections underline the importance of recognizing the socio-cultural meanings of pet ownership when designing pet-targeting health interventions.

Contextual factors additionally shaped the results. In this regard, compliance with guidelines, ratings, and regulations are shown to higher acceptance of infectious disease measures. Technical applications benefit from higher acceptance when they provide user-friendliness and technical quality. The acceptance of measures generally seem to be positively related to higher severity of the disease and an earlier stage of the disease. These findings illustrate that acceptance is not static but embedded in broader social contexts.

Taken together, these findings highlight that successful One Health policies require more than just technical solutions. They depend on an integrated understanding of veterinary expertise, economic and organizational design, and the behavioral and cultural factors shaping human-animal relations. Policies that are scientifically sound but fail to account for these factors risk not achieving widespread acceptance or successful implementation.

## Conclusion

The current evidence suggests that there is a diverse range of factors influencing the acceptance of disease prevention measures for pets, whereby the personal characteristics of concerned stakeholders resulted in being the largest category. It can be said that a broad, unambiguous, and transparent information distribution to the population by the inclusion of veterinarians, confident leadership, financial support in the implementation of measures, and accessible, uncomplicated measure implementation support the public acceptance of policy measures. Still, personal, animal-specific, and situational specificities do influence the acceptance of disease prevention measures and have to be acknowledged and included in the overall approach of the measure implementation.

Successful One Health policies, therefore, depend on an integrated understanding of veterinary, social, and behavioral contexts. Veterinary perspectives must be combined with insights into owners' attitudes, cultural norms, and broader societal values. Recognizing human behavioral drivers such as risk perception, trust in institutions, and fiscal aspects is essential for designing interventions that are not only scientifically appropriate but also achieve public acceptance. Embedding these dimensions into policy frameworks can help to ensure that One Health measures for companion animal disease prevention and combat are both effective and broadly supported.

It can be concluded that a variety of factors influence the acceptance of policy measures from the administrative as well as the population sides, and their interplay has to be considered in order to achieve successful implementation.

### Limitations

This study incorporates the maximum number of relevant studies possible, selected through a comprehensive and systematic search strategy. However, the literature search included only published articles and excluded government reports and other published sources, which could have introduced selective reporting and publication biases. In the following, the review does not address the complete scope of acceptance of policy measures on pets, and conclusions resulting from this review should be considered within the scope and methodology of the included studies.

Limitations in the methodology of the review include the fact that the titles and abstracts were screened, and data extraction and critical appraisal were conducted by a single reviewer.

Studies were excluded if they only marginally discussed disease prevention, management and combat measures for pets. Therefore, relevant studies were potentially excluded. Furthermore, qualitative findings are subject to common limitations such as social desirability, under-reporting, and reliance on memory.

### Implications for policy and practice

This review provides evidence that supports the approach of addressing the topic of measures to prevent infectious disease outbreaks in pets. Furthermore, it highlights the areas of policy and practice that need to be addressed. Including the aspect of acceptability in the planning and implementation of policy measures is crucial, and the portrayed categories can serve as a reference for the adaptation and successful implementation of measures. Companion animals are potential targets of infectious diseases and pose, through their close contact with humans, a special health threat. Steps should be taken to ensure preparedness in terms of legal and policy frameworks in order to intervene when necessary, taking into consideration concrete approaches to ensure broad public acceptance of policy measures. Instruments should compromise the direct influence on measure acceptance through policy tools, such as extensive information dispersion, close and transparent guidance through state institutions, easy access, and public funding of measures. However, they can also include more indirect elements, such as courses helping citizens to handle aggressive pets.

### Future research

Research on the acceptance of policy measures in disease prevention, management, and combat, especially in under-researched domains, such as the one of companion animals, remains important to help understand the practice, as well as inform successful policy and communication.

Research gaps relating to disease prevention for pets in developing countries, especially in sub-Saharan regions, should be further examined.

More research is needed to explore the different factors influencing the acceptance of measures and their backgrounds.

Furthermore, surveys and survey experiments could bring valuable knowledge to the understanding of the interplay between the factors influencing the acceptance of disease prevention measures for pets.

## Data Availability

The original contributions presented in the study are included in the article/Supplementary material, further inquiries can be directed to the corresponding author.

## Footnotes

1 Frequent travelling of a pet animal is hereby defined as at least once in 36 months (19).
